# Supplementary material for: SARS-CoV-2 Spike Protein Mutations and Escape from Antibodies: A Computational Model of Epitope Loss in Variants of Concern
Source: J Chem Inf Model. 2021 Sep 1;61(9):4687–700. doi: 10.1021/acs.jcim.1c00857 (PMC8479857; doi:10.1021/acs.jcim.1c00857)
Supplement: Supplementary file 1 — ci1c00857_si_001.pdf [file ci1c00857_si_001.pdf]

SUPPORTING INFORMATION

SARS-CoV-2 Spike Protein Mutations and Escape from Antibodies: a Computational Model of Epitope Loss in Variants of Concern

Alice Triveri,[1] Stefano A. Serapian,[1] Filippo Marchetti,[1] Filippo Doria,[1] Silvia Pavoni,[2] Fabrizio Cinquini,[3] Elisabetta Moroni,[4] Andrea Rasola,[5] Francesco Frigerio,[2] Giorgio Colombo[1,*]

[1)] University of Pavia, Department of Chemistry, via Taramelli 12, 27100 Pavia (Italy)

[2)] Department of Physical Chemistry, R&D Eni SpA, via Maritano 27, 20097 San Donato Milanese (Mi), Italy

[3)] Upstream & Technical Services – TECS/STES – Eni Spa, via Emilia 1, 20097 San Donato Milanese (Mi), Italy

[4)] Istituto di Scienze e Tecnologie Chimiche "Giulio Natta" - SCITEC, CNR Via Mario Bianco 9, 20131 Milano (Italy)

[5)] University of Padua, Department of Biomedical Sciences, viale G. Colombo 3, 35131 Padova (Italy)

* Corresponding Author: Giorgio Colombo, email: g.colombo@unipv.it

Table S1. The complete references to papers in which the various Abs targeting the Spike protein and its variants have been characterized and used for reference in the paper.

Anticorpo	PDB code	D614G	B.1.1.7	501Y.V2.noΔ	P.1	N439K	N501Y	PT188-EM
**REGN10987**	6XDG	Hansen[1]	Tada[2]	Tada[2]	Wang[3]	Thomson[4]	Luan[5]	
**REGN10933**	6XDG	Hansen[1]	Tada[2]	Tada[2]	Wang[3]	Thomson[4]	Luan[5]	
**LY-CoV555**	7L3N	Jones[6]	Wang[7]	Wang[7]		Thomson[4]	Luan[5]	
**S309**	7JX3/6WPT	Pinto[8]	Wang[7]	Wang[7]	Wang[3]	Thomson[4]	Luan[5]	
**C135**	7K8Z	Barnes[9]	Wang[7]	Wang[7]		Weisblum[10]		
**C144**	7K90	Barnes[9]		Martinez[11]	Weisblum[10]	Weisblum[10]		
**C121**	7K90	Barnes[9]		Martinez[11]	Wang[3]	Weisblum[10]		
**4A8**	7C2L	Chi[12]	Wang[13]	Wang[13]	Wang[14]			Andreano[15]
**DH1041**	7LAA	Gobeil[16]	Gobeil[16]	Gobeil[16]	Shen[17]			
**DH1043**	7LJR	Gobeil[16]	Gobeil[16]	Gobeil[16]	Shen[17]			
**DH1047**	7LD1	Gobeil[16]	Gobeil[16]	Gobeil[16]	Shen[17]			
**DH1050.1**	7LCN	Gobeil[16]	Gobeil[16]	Gobeil[16]	Shen[17]			
**S2M11**	7K43	Tortici[18]		Collier[19]	Collier[19]			
**COVA1-16**	7JMX	Wang[13]	Wang[13]	Wang[13]	Liu[20]		Wang[13]	
**B38**	7BZ5	Wu[21]	Shen[17]				Luan[5]	
**C002**	7K8T	Barnes[9]						
**CB6(LY-CoV016)**	7C01	Shi[22]	Wang[7]	Wang[7]	Wang[3]		Luan[5]	

1. Hansen, J.; Baum, A.; Pascal, K. E.; Russo, V.; Giordano, S.; Wloga, E.; Fulton, B. O.; Yan, Y.; Koon, K.; Patel, K.; Chung, K. M.; Hermann, A.; Ullman, E.; Cruz, J.; Rafique, A.; Huang, T.; Fairhurst, J.; Libertiny, C.; Malbec, M.; Lee, W. Y.; Welsh, R.; Farr, G.; Pennington, S.; Deshpande, D.; Cheng, J.; Watty, A.; Bouffard, P.; Babb, R.; Levenkova, N.; Chen, C.; Zhang, B.; Romero Hernandez, A.; Saotome, K.; Zhou, Y.; Franklin, M.; Sivapalasingam, S.; Lye, D. C.; Weston, S.; Logue, J.; Haupt, R.; Frieman, M.; Chen, G.; Olson, W.; Murphy, A. J.; Stahl, N.; Yancopoulos, G. D.; Kyratsous, C. A., Studies in humanized mice and convalescent humans yield a SARS-CoV-2 antibody cocktail. *Science* **2020,** *369* (6506), 1010-1014.

2. Tada, T.; Dcosta, B. M.; Zhou, H.; Vaill, A.; Kazmierski, W.; Landau, N. R., Decreased neutralization of SARS-CoV-2 global variants by therapeutic anti-spike protein monoclonal antibodies. *bioRxiv* **2021**.
3. Wang, P.; Casner, R. G.; Nair, M. S.; Wang, M.; Yu, J.; Cerutti, G.; Liu, L.; Kwong, P. D.; Huang, Y.; Shapiro, L.; Ho, D. D., Increased resistance of SARS-CoV-2 variant P.1 to antibody neutralization. *Cell Host Microbe* **2021,** *29* (5), 747-751.e4.
4. Thomson, E. C.; Rosen, L. E.; Shepherd, J. G.; Spreafico, R.; da Silva Filipe, A.; Wojcechowskyj, J. A.; Davis, C.; Piccoli, L.; Pascall, D. J.; Dillen, J.; Lytras, S.; Czudnochowski, N.; Shah, R.; Meury, M.; Jesudason, N.; De Marco, A.; Li, K.; Bassi, J.; O'Toole, A.; Pinto, D.; Colquhoun, R. M.; Culap, K.; Jackson, B.; Zatta, F.; Rambaut, A.; Jaconi, S.; Sreenu, V. B.; Nix, J.; Zhang, I.; Jarrett, R. F.; Glass, W. G.; Beltramello, M.; Nomikou, K.; Pizzuto, M.; Tong, L.; Cameroni, E.; Croll, T. I.; Johnson, N.; Di Iulio, J.; Wickenhagen, A.; Ceschi, A.; Harbison, A. M.; Mair, D.; Ferrari, P.; Smollett, K.; Sallusto, F.; Carmichael, S.; Garzoni, C.; Nichols, J.; Galli, M.; Hughes, J.; Riva, A.; Ho, A.; Schiuma, M.; Semple, M. G.; Openshaw, P. J. M.; Fadda, E.; Baillie, J. K.; Chodera, J. D.; Rihn, S. J.; Lycett, S. J.; Virgin, H. W.; Telenti, A.; Corti, D.; Robertson, D. L.; Snell, G.; Investigators, I. C.; Consortium, C.-G. U. C.-U., Circulating SARS-CoV-2 spike N439K variants maintain fitness while evading antibody-mediated immunity. *Cell* **2021,** *184* (5), 1171-1187.e20.
5. Luan, B.; Wang, H.; Huynh, T., Enhanced binding of the N501Y-mutated SARS-CoV-2 spike protein to the human ACE2 receptor: insights from molecular dynamics simulations. *FEBS Lett* **2021,** *595* (10), 1454-1461.
6. Jones, B. E.; Brown-Augsburger, P. L.; Corbett, K. S.; Westendorf, K.; Davies, J.; Cujec, T. P.; Wiethoff, C. M.; Blackbourne, J. L.; Heinz, B. A.; Foster, D.; Higgs, R. E.; Balasubramaniam, D.; Wang, L.; Bidshahri, R.; Kraft, L.; Hwang, Y.; Žentelis, S.; Jepson, K. R.; Goya, R.; Smith, M. A.; Collins, D. W.; Hinshaw, S. J.; Tycho, S. A.; Pellacani, D.; Xiang, P.; Muthuraman, K.; Sobhanifar, S.; Piper, M. H.; Triana, F. J.; Hendle, J.; Pustilnik, A.; Adams, A. C.; Berens, S. J.; Baric, R. S.; Martinez, D. R.; Cross, R. W.; Geisbert, T. W.; Borisevich, V.; Abiona, O.; Belli, H. M.; de Vries, M.; Mohamed, A.; Dittmann, M.; Samanovic, M.; Mulligan, M. J.; Goldsmith, J. A.; Hsieh, C. L.; Johnson, N. V.; Wrapp, D.; McLellan, J. S.; Barnhart, B. C.; Graham, B. S.; Mascola, J. R.; Hansen, C. L.; Falconer, E., LY-CoV555, a rapidly isolated potent neutralizing antibody, provides protection in a non-human primate model of SARS-CoV-2 infection. *bioRxiv* **2020**.
7. Wang, L.; Zhou, T.; Zhang, Y.; Yang, E. S.; Schramm, C. A.; Shi, W.; Pegu, A.; Oloniniyi, O. K.; Henry, A. R.; Darko, S.; Narpala, S. R.; Hatcher, C.; Martinez, D. R.; Tsybovsky, Y.; Phung, E.; Abiona, O. M.; Antia, A.; Cale, E. M.; Chang, L. A.; Choe, M.; Corbett, K. S.; Davis, R. L.; DiPiazza, A. T.; Gordon, I. J.; Helmold Hait, S.; Hermanus, T.; Kgagudi, P.; Laboune, F.; Leung, K.; Liu, T.; Mason, R. D.; Nazzari, A. F.; Novik, L.; O'Connell, S.; O'Dell, S.; Olia, A. S.; Schmidt, S. D.; Stephens, T.; Stringham, C. D.; Talana, C. A.; Teng, I. T.; Wagner, D. A.; Widge, A. T.; Zhang, B.; Roederer, M.; Ledgerwood, J. E.; Ruckwardt, T. J.; Gaudinski, M. R.; Moore, P. L.; Doria-Rose, N. A.; Baric, R. S.; Graham, B. S.; McDermott, A. B.; Douek, D. C.; Kwong, P. D.; Mascola, J. R.; Sullivan, N. J.; Misasi, J., Ultrapotent antibodies against diverse and highly transmissible SARS-CoV-2 variants. *Science* **2021**.
8. Pinto, D.; Park, Y. J.; Beltramello, M.; Walls, A. C.; Tortorici, M. A.; Bianchi, S.; Jaconi, S.; Culap, K.; Zatta, F.; De Marco, A.; Peter, A.; Guarino, B.; Spreafico, R.; Cameroni, E.; Case, J. B.; Chen, R. E.; Havenar-Daughton, C.; Snell, G.; Telenti, A.; Virgin, H. W.; Lanzavecchia, A.; Diamond, M. S.; Fink, K.; Veesler, D.; Corti, D., Cross-neutralization of SARS-CoV-2 by a human monoclonal SARS-CoV antibody. *Nature* **2020,** *583* (7815), 290-295.
9. Barnes, C. O.; Jette, C. A.; Abernathy, M. E.; Dam, K. A.; Esswein, S. R.; Gristick, H. B.; Malyutin, A. G.; Sharaf, N. G.; Huey-Tubman, K. E.; Lee, Y. E.; Robbiani, D. F.; Nussenzweig, M.

C.; West, A. P.; Bjorkman, P. J., SARS-CoV-2 neutralizing antibody structures inform therapeutic strategies. *Nature* **2020,** *588* (7839), 682-687.
10. Weisblum, Y.; Schmidt, F.; Zhang, F.; DaSilva, J.; Poston, D.; Lorenzi, J. C.; Muecksch, F.; Rutkowska, M.; Hoffmann, H. H.; Michailidis, E.; Gaebler, C.; Agudelo, M.; Cho, A.; Wang, Z.; Gazumyan, A.; Cipolla, M.; Luchsinger, L.; Hillyer, C. D.; Caskey, M.; Robbiani, D. F.; Rice, C. M.; Nussenzweig, M. C.; Hatziioannou, T.; Bieniasz, P. D., Escape from neutralizing antibodies by SARS-CoV-2 spike protein variants. *Elife* **2020,** *9*.
11. Martinez, D. R.; Schaefer, A.; Leist, S. R.; Gully, K.; Feng, J. Y.; Bunyan, E.; Porter, D. P.; Cihlar, T.; Montgomery, S. A.; Baric, R. S.; Nussenzweig, M. C.; Sheahan, T. P., Early therapy with remdesivir and antibody combinations improves COVID-19 disease in mice. *bioRxiv* **2021**.
12. Chi, X.; Yan, R.; Zhang, J.; Zhang, G.; Zhang, Y.; Hao, M.; Zhang, Z.; Fan, P.; Dong, Y.; Yang, Y.; Chen, Z.; Guo, Y.; Li, Y.; Song, X.; Chen, Y.; Xia, L.; Fu, L.; Hou, L.; Xu, J.; Yu, C.; Li, J.; Zhou, Q.; Chen, W., A neutralizing human antibody binds to the N-terminal domain of the Spike protein of SARS-CoV-2. *Science* **2020,** *369* (6504), 650-655.
13. Wang, P.; Nair, M. S.; Liu, L.; Iketani, S.; Luo, Y.; Guo, Y.; Wang, M.; Yu, J.; Zhang, B.; Kwong, P. D.; Graham, B. S.; Mascola, J. R.; Chang, J. Y.; Yin, M. T.; Sobieszczyk, M.; Kyratsous, C. A.; Shapiro, L.; Sheng, Z.; Huang, Y.; Ho, D. D., Antibody resistance of SARS-CoV-2 variants B.1.351 and B.1.1.7. *Nature* **2021,** *593* (7857), 130-135.
14. Wang, R.; Zhang, Q.; Ge, J.; Ren, W.; Zhang, R.; Lan, J.; Ju, B.; Su, B.; Yu, F.; Chen, P.; Liao, H.; Feng, Y.; Li, X.; Shi, X.; Zhang, Z.; Zhang, F.; Ding, Q.; Zhang, T.; Wang, X.; Zhang, L., Analysis of SARS-CoV-2 variant mutations reveals neutralization escape mechanisms and the ability to use ACE2 receptors from additional species. *Immunity* **2021**.
15. Andreano, E.; Nicastri, E.; Paciello, I.; Pileri, P.; Manganaro, N.; Piccini, G.; Manenti, A.; Pantano, E.; Kabanova, A.; Troisi, M.; Vacca, F.; Cardamone, D.; De Santi, C.; Torres, J. L.; Ozorowski, G.; Benincasa, L.; Jang, H.; Di Genova, C.; Depau, L.; Brunetti, J.; Agrati, C.; Capobianchi, M. R.; Castilletti, C.; Emiliozzi, A.; Fabbiani, M.; Montagnani, F.; Bracci, L.; Sautto, G.; Ross, T. M.; Montomoli, E.; Temperton, N.; Ward, A. B.; Sala, C.; Ippolito, G.; Rappuoli, R., Extremely potent human monoclonal antibodies from COVID-19 convalescent patients. *Cell* **2021,** *184* (7), 1821-1835.e16.
16. Gobeil, S. M.; Janowska, K.; McDowell, S.; Mansouri, K.; Parks, R.; Stalls, V.; Kopp, M. F.; Manne, K.; Li, D.; Wiehe, K.; Saunders, K. O.; Edwards, R. J.; Korber, B.; Haynes, B. F.; Henderson, R.; Acharya, P., Effect of natural mutations of SARS-CoV-2 on spike structure, conformation, and antigenicity. *Science* **2021**.
17. Shen, X.; Tang, H.; McDanal, C.; Wagh, K.; Fischer, W.; Theiler, J.; Yoon, H.; Li, D.; Haynes, B. F.; Sanders, K. O.; Gnanakaran, S.; Hengartner, N.; Pajon, R.; Smith, G.; Glenn, G. M.; Korber, B.; Montefiori, D. C., SARS-CoV-2 variant B.1.1.7 is susceptible to neutralizing antibodies elicited by ancestral spike vaccines. *Cell Host Microbe* **2021,** *29* (4), 529-539.e3.
18. Tortorici, M. A.; Beltramello, M.; Lempp, F. A.; Pinto, D.; Dang, H. V.; Rosen, L. E.; McCallum, M.; Bowen, J.; Minola, A.; Jaconi, S.; Zatta, F.; De Marco, A.; Guarino, B.; Bianchi, S.; Lauron, E. J.; Tucker, H.; Zhou, J.; Peter, A.; Havenar-Daughton, C.; Wojcechowskyj, J. A.; Case, J. B.; Chen, R. E.; Kaiser, H.; Montiel-Ruiz, M.; Meury, M.; Czudnochowski, N.; Spreafico, R.; Dillen, J.; Ng, C.; Sprugasci, N.; Culap, K.; Benigni, F.; Abdelnabi, R.; Foo, S. C.; Schmid, M. A.; Cameroni, E.; Riva, A.; Gabrieli, A.; Galli, M.; Pizzuto, M. S.; Neyts, J.; Diamond, M. S.; Virgin, H. W.; Snell, G.; Corti, D.; Fink, K.; Veesler, D., Ultrapotent human antibodies protect against SARS-CoV-2 challenge via multiple mechanisms. *Science* **2020,** *370* (6519), 950-957.
19. Collier, D. A.; De Marco, A.; Ferreira, I. A. T. M.; Meng, B.; Datir, R. P.; Walls, A. C.; Kemp, S. A.; Bassi, J.; Pinto, D.; Silacci-Fregni, C.; Bianchi, S.; Tortorici, M. A.; Bowen, J.; Culap, K.; Jaconi, S.; Cameroni, E.; Snell, G.; Pizzuto, M. S.; Pellanda, A. F.; Garzoni, C.; Riva, A.; Elmer, A.;

Kingston, N.; Graves, B.; McCoy, L. E.; Smith, K. G. C.; Bradley, J. R.; Temperton, N.; Ceron-Gutierrez, L.; Barcenas-Morales, G.; Harvey, W.; Virgin, H. W.; Lanzavecchia, A.; Piccoli, L.; Doffinger, R.; Wills, M.; Veesler, D.; Corti, D.; Gupta, R. K.; Collaboration, C.-N. B. C.-.; Consortium, C.-G. U. C.-U., Sensitivity of SARS-CoV-2 B.1.1.7 to mRNA vaccine-elicited antibodies. *Nature* **2021,** *593* (7857), 136-141.
20. Liu, H.; Wu, N. C.; Yuan, M.; Bangaru, S.; Torres, J. L.; Caniels, T. G.; van Schooten, J.; Zhu, X.; Lee, C. D.; Brouwer, P. J. M.; van Gils, M. J.; Sanders, R. W.; Ward, A. B.; Wilson, I. A., Cross-Neutralization of a SARS-CoV-2 Antibody to a Functionally Conserved Site Is Mediated by Avidity. *Immunity* **2020,** *53* (6), 1272-1280.e5.
21. Wu, Y.; Wang, F.; Shen, C.; Peng, W.; Li, D.; Zhao, C.; Li, Z.; Li, S.; Bi, Y.; Yang, Y.; Gong, Y.; Xiao, H.; Fan, Z.; Tan, S.; Wu, G.; Tan, W.; Lu, X.; Fan, C.; Wang, Q.; Liu, Y.; Zhang, C.; Qi, J.; Gao, G. F.; Gao, F.; Liu, L., A noncompeting pair of human neutralizing antibodies block COVID-19 virus binding to its receptor ACE2. *Science* **2020,** *368* (6496), 1274-1278.
22. Shi, R.; Shan, C.; Duan, X.; Chen, Z.; Liu, P.; Song, J.; Song, T.; Bi, X.; Han, C.; Wu, L.; Gao, G.; Hu, X.; Zhang, Y.; Tong, Z.; Huang, W.; Liu, W. J.; Wu, G.; Zhang, B.; Wang, L.; Qi, J.; Feng, H.; Wang, F. S.; Wang, Q.; Gao, G. F.; Yuan, Z.; Yan, J., A human neutralizing antibody targets the receptor-binding site of SARS-CoV-2. *Nature* **2020,** *584* (7819), 120-124.

Table S2. The residues that are part of the experimentally determined epitopes and of the predicted epitopes on the various Spike variants. The numbering of the residues refers to that reported in UNIPROT entry P0DTC2. https://www.uniprot.org/uniprot/P0DTC2.

Antibody	Experimental	D614G	N439K	501Y.V2.noΔ	B.1.1.7	P.1	N501Y	PT188-EM
**4A8**	143-148,150,152,245-250,256-257	143-147,245-246,249	144-147,150,152,257	no	143,145,147-148,245	143,145-148,245,248,250,256	no	147,150,152
**DH1050.1**	140-146,148-152,156-159,244-245	140,143-146,156,158-159,245	140-141,144-146,149-152,159,244	no	142-143,145,148-149,151,157,244-245	142-143,145-146,148-149,156-157,244-245	no	149-152
**REGN10987**	345-346,439-441,443-447,449,490-500	439,441,443-447,449,491-498	no	345-346,439,441,443,490-491,494-497	439-441,443-447,449,490,492,493-500	491	no	439-440
**REGN10933**	403,405,417,421,453,455-456,473,475-478,484-490,492-494,498	403,405,421,453,456,473,476,478,485,487,489,492-494,498	484,486-487	403,405,453,477,484-485,489-490,494	403,421,453,456,473,475,477-478,485-486,488-490,492-494,498	421	453,473,475-478,487	403,406
**S309**	333-341,343-345,354,356-361,441,444,509	334-341,354,356-361,441,444,509	337,340	333-335,337-338,340,344-345,354,358,360,441,509	334-338,340,354,356-361,441,509	335	336,354,356,358-359,361,509	357,359-360,509
**LY-CoV555**	351,449-450,452-453,456,470,472,478,481-490,492-496	351,449-450,452-453,456,472,478,485,487,489,492-496	482-484,486-487	351,452-453,470,472,483-485,489-490,494-496	351,449,452-453,456,470,472,478,482,485-486,488-490,492-496	no	453,470,472,478,482-483,487	no
**C135**	341-346,438-442,490	341,438-439,441-442	no	344-346,439,441-442,490	448-442,490	438	438	439-440
**C144**	342-343,367,370-374,417,436,440,449-450,452,455-456,472,483-487,498	367,371,373-374,436,449-450,456,472,485,487,498	483-484,486-487	367,371-374,436,472,483-485	372-374,449,455-456,472,485-486,498	367,370,373-374	436,472,483,487	436
**C121**	444,449,483-490,492-496	444,449,485,487,489,492-496	483-484,486-487	483-485,489-490,494-496	449,485-486,488-490,492-496	no	483,487	no
**DH1041**	351,446-452,470-472,480-486,490-494,496	351,446-452,471-472,480,485,491-494,496	480,482-484,486	351,448,451-452,470,472,483-485,490-491,494,496	351,446-449,451-452,470-472,482,485-486,490,492-494,496	491	470-472,480,482-483	no
**DH1043**	351,446-447,449-450,452,456,470,472,478-494	351,446-447,449-450,452,456,472,478,480,485,487,489,491-494	479-480,482-484,486-487	351,452,470,472,483-485,489-491,494	351,446-447,449,452,456,470,472,478-479,482,485-486,488-490,492-494	491	470,472,478-480,482-483,487	no
**DH1047**	369-372,374-380,383-384,404-405,407-409,414-416,435,499,501-505	369,371,374-380,383-384,404-405,407-409,414-416,435,503,505	no	369,371-372,374-379,383-384,404,407,409,414-416,435	369,372,374-379,383,405,407-408,414-416,435,499,501-503,505	370,374,377,380,404,416	375,384,416	380,404-405,407-409,435,501,503-505
**S2M11**	342-343,367-368,371-373,436,440-441,446-447,449,452,455-456,484-487,489-490,492-496,498	367,371,373-374,436,441,446-447,449,452,456,485,487,489,492-496,498	484,486-487	367-368,371-374,436,441,452,484-485,489-490,494-496	368,372-374,440-441,446-447,449,452,455-456,485-486,489-490,492-496,498	367-368,373-374	368,436,487	436,440
**COVA1-16**	368-369,371,374-385,408-409,412-416,427-429	368-369,371,374-381,384,408-409,412,414-416,428	no	368-369,371,374-379,382-385,409,412-416,427-429	368-369,374-379,381-383,385,408,414-416	368,374,377,380-382,412,416	368,375,384,416	380-381,408-409,429
**B38**	403,405-406,409,415-417,420-421,449,453,455-460,473-477,484,486-498,500-503,505	403,405-406,409,415-416,420-421,449,453,456,473-474,476,487,489,491-498,503,505	484,486-487	403,406,409,420,453,474,477,484,489-491,494-497	403,405,415,421,449,453,455,457-460,473-476,484,487-480,501-498,500-502,505	416,420-421,460,491	416,453,473-477,487	403,405-406,409,501,505
**C002**	444,447,449-450,452,470,473,481-490,492-494	444,447,449-450,452,473,485,487,489,492-494	482-484,486-487	452,470,483-485,489-490,494	447,449,452,470,473,482,485-486,488-490,492-495	no	470,473,482-483,487	no
**CB6 (LY-CoV016)**	403,405-406,408-409,415-417,420-421,453,455-460,473-477,486-487,489,493-495,500-505	403,405-406,408-409,415-416,420-421,453,456,473-474,476,487,489,493-495,503,505	486-487	403,406,409,415-416,420,453,474,477,489,494-495	403,405,408,415,421,453,455-459,473-475,478,486,489,493-495,500-503,505	416,420-421,460	416,453,473-477,487	403,405-406,408-409,501,503-505
